# Primary hepatic ectopic pregnancy in a patient with polycystic ovary syndrome: A case report

**DOI:** 10.1097/MD.0000000000019649

**Published:** 2020-03-27

**Authors:** Ning Zhang, Linqing Yang, Yunfei Wang, Xiaoyu Li, Chao Zhang, Jing Xu

**Affiliations:** a School of Clinical Medicine, Jining Medical University; b Departments of Gynecology; c Department of Otolaryngology-Head and Neck Surgery, The Affiliated Hospital of Jining Medical University, Jining, Shandong Province, China.

**Keywords:** abdominal pregnancy, case report, ectopic pregnancy, hepatic ectopic pregnancy, laparoscopic, laparotomy

## Abstract

**Rationale:**

Hepatic ectopic pregnancy is an extremely rare ectopic pregnancy. This study aimed to report a case of primary hepatic pregnancy in a patient with polycystic syndrome.

**Patient concerns:**

A 30-year-old woman presented with vaginal bleeding after 63 days of amenorrhea.

**Diagnosis:**

The patient was initially diagnosed with liver ectopic pregnancy using abdominal ultrasound and abdominal computed tomography (CT).

**Interventions:**

The patient underwent laparoscopic exploration to reconfirm the gestational sac in the liver and abdominal surgery to remove liver gestation. The postoperative review of abdominal CT and the level of serum human chorionic gonadotropin (hCG) was performed.

**Outcomes:**

The postoperative pathological examination revealed a fluffy tissue in the liver tissue and a blood clot. The patient's vital signs were normal, and she was advised regular follow-up after discharge from the hospital. One month later, the serum hCG level reduced to 0.32 mIU/mL (reference range 0–5 mIU/mL).

**Lessons:**

If the level of beta-human chorionic gonadotropin (β-HCG) is higher than normal in women of childbearing age and no gestational sac is found in the uterine cavity, the location of pregnancy and gestational sac should be positively confirmed. Also, the possibility of ectopic pregnancy in the abdominal cavity should be considered, and the relevant imaging and biochemical examinations should be improved to avoid delay in diagnosis and treatment.

## 1 Introduction

Ectopic pregnancy refers to the attachment of the zygote to the extrauterine cavity. It often occurs in fallopian tubes, ovaries, broad ligaments, and so on, but seldom in the abdominal cavity such as liver, spleen, or peritoneum.^[1]^ It has an incidence of 1.4% and a mortality rate of 5.1/1000, which is 7.7 times higher than that of nonabdominal ectopic pregnancy.^[2]^ If the hepatic ectopic pregnancy cannot be diagnosed and treated in time, the patient suffers a hemorrhage in the abdominal cavity. This study described the case of a patient with liver pregnancy.

## 2 Case report

A 30-year-old pregnant patient (gravida 1, para 1) presented at the gynecologic department with vaginal bleeding after 63 days of amenorrhea, but no abdominal/pelvic pain. The self-test urine pregnancy test was weakly positive after menopause for 35 days. The serum human chorionic gonadotropin (hCG) level showed a progressive elevation. The ultrasound (US) scan showed no gestational sac in the uterine cavity and no abnormalities in the double attachment. After menopause for 56 days, the serum hCG level was 8147 IU/L, with complaints of abdominal pain and anal bulge.

**Figure 1:**
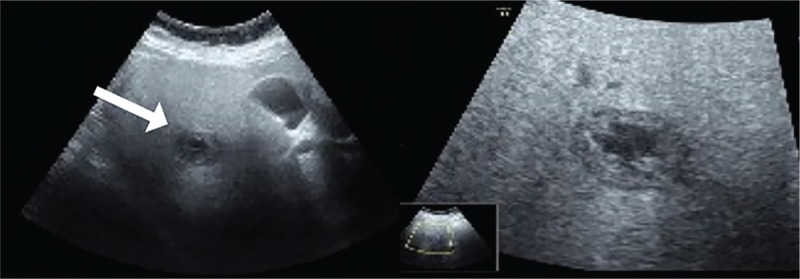
Abdominal ultrasound examination showed uneven fatty liver and a cystic echo mass (white arrow) measuring 30 × 19 × 25 mm^3^, with a hypoechoic nucleus, attached to the inferior surface of the right hepatic lobe.

**Figure 2:**
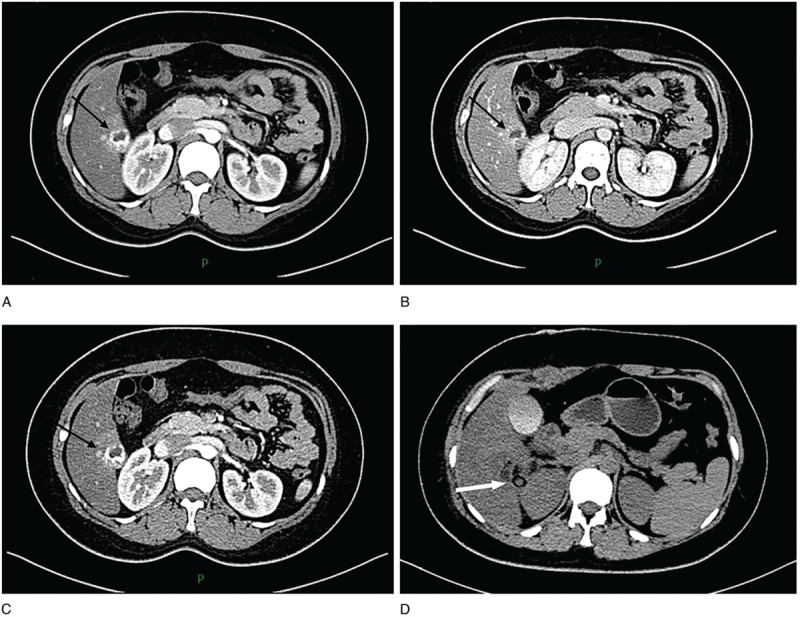
(A–C) Abdominal computed tomography performed with contrast enhancement. The arterial phase revealed a round lesion, with a peripheral portion hypervascularized with significantly increased density. The main axis of the lesion was 29 mm (black arrow). (D) Abdominal computed tomography scan showed the drainage tube drawn from the surgical field after the surgery (white arrow).

A week later, her serum hCG level increased to17,193 IU/L. Her blood pressure was 132/80 mm Hg. She had a pulse rate of 97 beats/min, respiratory rate of 20 breaths/min, temperature of 36.7°C, body mass index of 27.94 kg/m^2^, and hemoglobin level of 131 g/L. No tenderness or rebound was observed in the upper abdomen. The US scan revealed a conspicuous amount of echogenic fluid behind the uterine cavity, a mixed echogenic mass in the hepatic area, and multiple cystic changes in bilateral ovaries (Fig. 1). A subsequent computed tomography (CT) of the abdomen with intravenous contrast showed fatty liver and a lesion of 29 × 23 × 25 mm^3^, which was uneven and peripherally vascularized in the arterial phase obviously, in the hepatic segment VI (Fig. 2). This explained the previous negative US findings. The clinician's initial diagnosis was suspected liver ectopic pregnancy and polycystic ovary syndrome.

The laparoscopic exploration was performed by an expert gynecologic surgeon, which revealed pelvic cavity hemorrhage and a round 25-mm ectopic pregnancy lesion in the hepatic segment IV. The uterus, ovaries, and fallopian tubes were normal. Also, gastrointestinal surgery consultation indicated that the intestines, stomach, and spleen were normal. The laparoscopic exploration was stopped, and laparotomy was performed by expert hepatobiliary-pancreatic surgeons, considering the possibility of uncontrolled bleeding after continued surgery.

**Figure 3:**
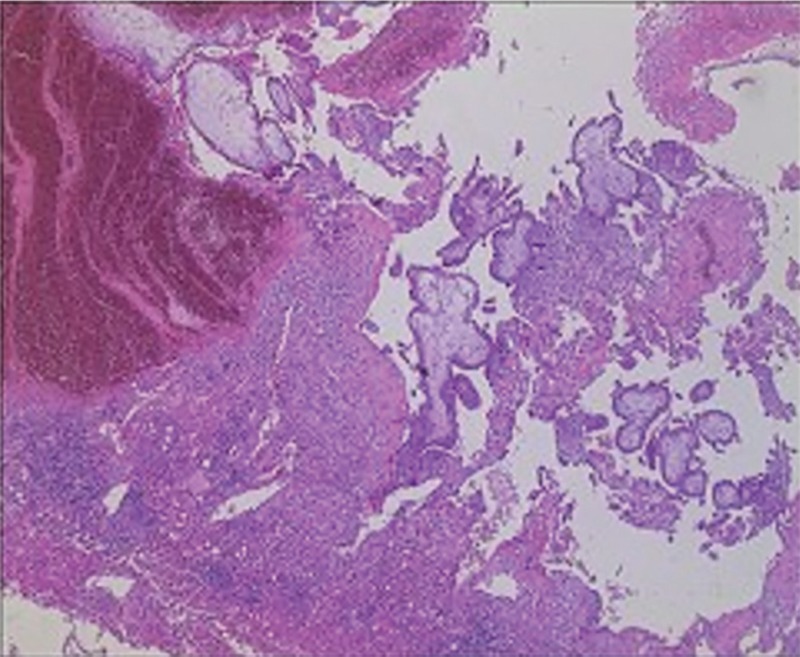
Histologic examination (hematoxylin and eosin staining, original magnification ×40) showed chorionic villi infiltrating into the liver tissue.

The laparotomy was performed through a right subcostal incision of 20 cm in length under general anesthesia. The right lobe of the liver was fully exposed, and the first hepatic hilum was ligated temporarily. The clamp method was used to completely remove the ectopic pregnancy lesion, and the distance from the cutting edge to the pregnancy lesion was 1 cm. No active bleeding or bile leakage was found in the section of the liver. A drainage tube was placed in the right hepatic section. The pathological examination of the excised tissue indicated a hepatic ectopic pregnancy (Fig. 3).

The patient's serum hCG level dropped to 3696 and 1071.67 IU/L, respectively, 24 and 72 after the surgery. The CT of the abdomen showed that the patient gradually recovered after the surgery (Fig. 2). She was discharged on postoperative day 8.

**Figure 4:**
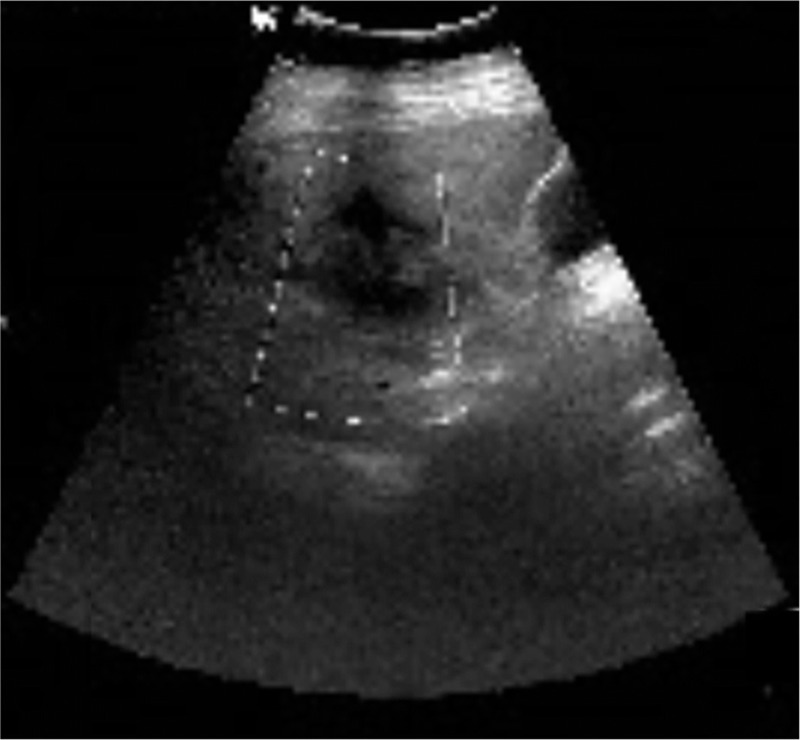
Ultrasound image of the liver showed uneven fatty liver and an inhomogeneous echo zone in the surgical area of the liver, with no blood flow signal.

The outpatient doctor's follow-up revealed that the patient's serum hCG level reduced to 0.32 mIU/mL (reference range: 0–5 mIU/mL) 36 days after the surgery. Liver US revealed postoperative imaging changes in the liver (Fig. 4). The patient returned to the prepregnancy status.

## 3 Discussion

The incidence of hepatic ectopic pregnancy is lower than that of ectopic pregnancy in other locations. Only 41 cases of primary hepatic ectopic pregnancy have been reported since 1952,^[3]^ based on the Medline database search. As the majority of abdominal pregnancies are implanted in the pelvis, it is rare to find ectopic implantation on the peritoneal wall of the abdomen.^[4]^ Although hepatic ectopic pregnancy is an exceptionally rare type of abdominal pregnancy that commonly remains undiagnosed, the possibility of an unusual location must always be a part of the practitioner's differential diagnosis.^[5]^ The mortality rate of hepatic ectopic pregnancy is 5 to 7 times higher than the rate observed in tubal ectopic pregnancies.^[6]^ Most patients with hepatic pregnancy have a history of wearing intrauterine devices, and implantation occurs on the lower surface of the right lobe of the liver.^[7]^ In the present case, the zygote also implanted on the right lobe, and the patient had no history of wearing intrauterine devices. Some literature raised the opinion that polycystic ovary syndrome is positively related to the occurrence of ectopic pregnancy after controlled ovarian hyperstimulation.^[8,9]^ This study reported 1 case of liver ectopic pregnancy in patients with polycystic ovary without controlled ovarian hyperstimulation. Therefore, further research is needed on the risk of ectopic pregnancy in women with polycystic ovary in the absence of controlled ovarian hyperstimulation.

There is no clear evidence to explain the mechanism of liver pregnancy.^[10]^ According to the literature, this mechanism can be explained by the mechanism of ovarian cancer spread and metastasis.^[4,11]^ Abdominal pregnancy is when the fertilized egg does not enter the uterine cavity through the peristalsis of the fallopian tube, but enters the abdominal cavity with the peristaltic and respiratory movements of the intestine.^[4]^ With the fluid flow in the abdominal cavity, it then grows on the dense surface of blood vessels, such as the surface of the liver and spleen.^[7,12]^ Probably fatty liver and high vascularity of the hepatic parenchyma have the same effect, promoting zygote implantation on the surface of the liver and making the initial attachment in an ectopic pregnancy feasible.^[12,13]^ However, due to the effect of gravity, the fertilized eggs will swim to the lowest part of the abdominal cavity. When the supine position is located, the lower surface of the right lobe of the liver is the lowest position of the abdominal cavity. It explains that the location of the ectopic pregnancy of the liver often appears on the lower surface of the right lobe of the liver.^[4,12,14–17]^

The diagnosis of ectopic pregnancy is not simple, but the application of imaging technology can not only reduce misdiagnosis but also improve diagnosis efficiency.^[4,18]^ An abdominal US should be performed to hunt for abdominal implantation sites after a normal pelvic transvaginal US in a suspected ectopic pregnancy. US can detect fetal heartbeats, which is not only safe but also convenient for patients.^[18,19]^ However, due to the thickness of the abdominal fat layer and gas in the intestine of the patient,^[13]^ the application of the US will also be limited. CT and magnetic resonance imaging (MRI) can reflect the tissue structure near the gestational sac, which can assist in the subsequent choice of treatment compared with US.^[20]^ CT with intravenous contrast enhancement can detect lesions by distinguishing density.^[4,20]^ MRI can be used to diagnose ectopic pregnancy, and contrast-enhancing effects can show ring-shaped high-signal intensity in ectopic pregnancy tissues.^[21,22]^ Therefore, US, CT, and MRI can provide reference and evaluation value for the diagnosis and treatment of ectopic liver pregnancy.

The choices of treatment for ectopic pregnancy are expectant treatment (close monitoring), drug therapy, and surgery.^[23]^ Although the surgical excision of ectopic pregnancies is safe, certain risks still remain.^[24]^ They include complications arising from the administration of a general anesthetic and injury to major abdominal structures (bowel and major abdominal vessels).^[24]^ Due to the increased risk of maternal death, immediate surgical laparotomy and hepatectomy are commonly performed in traditional primary liver pregnancy management. The gestational sac diameter of more than 3.5 cm is a relative contraindication for methotrexate treatment.^[25]^ Drug treatment can be considered for the patient with suspected liver pregnancy to avoid the risk of surgery, pending further clinical data support.^[26,27]^ In the present case, hepatic ectopic pregnancy was suspected before surgery, and a combination of laparoscopic surgery and open surgery approach was used to safely remove the liver gestational sac and reduce the serum hCG level to normal range.

The diagnosis and treatment of these unusual abdominal pregnancies present both diagnostic and therapeutic dilemmas.^[28]^ Therefore, the key to the successful management of an unknown-location pregnancy is high suspicion. Whether it is laparoscopic or open surgery, the postoperative serum hCG level is a common observation indicator and also one of the determinants of using methotrexate after the surgery.^[29]^ The data to support the conservative treatment used in the present case are still insufficient, and surgical treatment might be the first choice. Regardless of laparoscopic or open surgery, a surgical path that suits the patient's condition should be chosen. In conclusion, gynecologists should consider the possibility of hepatic implantation when faced with such a clinical presentation, although it is extremely rare.

## Acknowledgment

The authors would like to thank our department colleagues and the devotion of this patient, and the patient has signed the informed consent form.

## Author contributions

**Data curation:** Yunfei Wang, Chao Zhang.

**Investigation:** Chao Zhang.

**Project administration:** Jing Xu.

**Writing – original draft:** Ning Zhang, Linqing Yang, Chao Zhang, Xiaoyu Li, Jing Xu.

**Writing – review & editing:** Xiaoyu Li, Jing Xu.

Xiaoyu Li orcid: 0000-0002-0221-2336.
